# Community use of face masks and similar barriers to prevent respiratory illness such as COVID-19: a rapid scoping review

**DOI:** 10.2807/1560-7917.ES.2020.25.49.2000725

**Published:** 2020-12-10

**Authors:** Julii Brainard, Natalia R. Jones, Iain R Lake, Lee Hooper, Paul R Hunter

**Affiliations:** 1 The Norwich School of Medicine, University of East Anglia, Norwich, Norfolk, United Kingdom; 2 School of Environmental Sciences, University of East Anglia, Norwich, Norfolk, United Kingdom

**Keywords:** coronavirus, face mask, influenza-like-illness, Hajj, respiratory infection

## Abstract

**Background:**

Evidence for face-mask wearing in the community to protect against respiratory disease is unclear.

**Aim:**

To assess effectiveness of wearing face masks in the community to prevent respiratory disease, and recommend improvements to this evidence base.

**Methods:**

We systematically searched Scopus, Embase and MEDLINE for studies evaluating respiratory disease incidence after face-mask wearing (or not). Narrative synthesis and random-effects meta-analysis of attack rates for primary and secondary prevention were performed, subgrouped by design, setting, face barrier type, and who wore the mask. Preferred outcome was influenza-like illness. Grading of Recommendations, Assessment, Development and Evaluations (GRADE) quality assessment was undertaken and evidence base deficits described.

**Results:**

33 studies (12 randomised control trials (RCTs)) were included. Mask wearing reduced primary infection by 6% (odds ratio (OR): 0.94; 95% CI: 0.75–1.19 for RCTs) to 61% (OR: 0.85; 95% CI: 0.32–2.27; OR: 0.39; 95% CI: 0.18–0.84 and OR: 0.61; 95% CI: 0.45–0.85 for cohort, case–control and cross-sectional studies respectively). RCTs suggested lowest secondary attack rates when both well and ill household members wore masks (OR: 0.81; 95% CI: 0.48–1.37). While RCTs might underestimate effects due to poor compliance and controls wearing masks, observational studies likely overestimate effects, as mask wearing might be associated with other risk-averse behaviours. GRADE was low or very low quality.

**Conclusion:**

Wearing face masks may reduce primary respiratory infection risk, probably by 6–15%. It is important to balance evidence from RCTs and observational studies when their conclusions widely differ and both are at risk of significant bias. COVID-19-specific studies are required.

## Introduction

On 30 January 2020 the World Health Organization (WHO) declared a Public Health Emergency of International Concern (PHEIC) in response to the emergence of a novel coronavirus in Wuhan, China [1]. On 11 March 2020 the WHO declared the coronavirus disease (COVID-19) epidemic to be a pandemic [2]. By the end of June 2020 nearly 500,000 global deaths had been linked to COVID-19 [3]. It is not clear when this outbreak will abate.

Among other advice widely sought by the public in response to this outbreak, was whether wearing face coverings, especially medical-grade coverings (e.g. masks, goggles or similar) might reduce the risk of catching or transmitting disease [4], particularly in domestic and public places. Sales of inexpensive face mask products soared following the PHEIC declaration, leading to potential shortages for social care and healthcare workers [5-10]. Previous systematic reviews on the efficacy and effectiveness of using face masks in community settings assessed face masks combined with other personal protection measures [11-13] or mixed healthcare workers with non-healthcare workers [12,14-16]. Those that specifically examined community use had focused only on randomised control trials (RCTs) [17,18]. Overall, the reviews had mixed conclusions about community settings: that face masks were highly effective [12,16], definitely effective [14,19], may be effective for protection [17,18,20] or did not have a statistically significant effect [12]. There has been near consensus that the evidence base is inadequate [11,14,17-20].

In early 2020 we responded to this information demand by undertaking a rapid scoping review using systematic review methods to evaluate evidence that might indicate the effectiveness of wearing face masks in the community in relation to the transmission of respiratory disease. This review therefore considers the quality of the evidence for these outcomes and produces recommendations on how to improve this evidence base.

## Methods

### Review aims

We aimed to assess the effectiveness of wearing a face barrier (mask, goggles, shield, veil) in community settings to prevent transmission of respiratory illness, such as from coronaviruses, rhinoviruses, influenza viruses or tuberculosis, and recommend how to improve this evidence base. We use the words mask and face mask interchangeably as umbrella terms for diverse facial coverings that may cover any combination of mouth, nose and/or eyes.

### Search strategy

Two recent literature reviews [12,18] were consulted to find 11 exemplar studies [21-31] that met our eligibility criteria. We designed search strategies that were sensitive enough to find these exemplar studies and similar research, yet specific enough exclude most irrelevant records. The bibliographic databases Scopus, Embase and Medline were searched with the phrases in the Box. We read other systematic reviews [11,12,14,16-20] on similar non-pharmaceutical practices to look for any missing primary studies.

Box **Bibliographic database search phrases**

**Scopus**

TITLE-ABS-KEY (

(facemask? OR “facemasks?” OR mask? OR goggle? OR faceshield? OR respirator OR respirators)

AND

(influenza OR flu OR sars OR tuberculosis or mers OR coronav* OR “cov” OR COVID* OR respiratory-syndrome OR wuhan or “ncov”)

)

AND

( LIMIT-TO ( SUBJAREA , “MEDI” ) OR LIMIT-TO ( SUBJAREA , “NURS” ) OR LIMIT-TO ( SUBJAREA , “IMMU” ) )

**Embase and Medline via OVID**

[(facemask* OR “face-mask*” OR mask* OR goggle* OR face-shield* OR respirator OR respirators).kw,ti,ab.]

and

[(influenza OR flu OR sars or tuberculosis OR mers or coronav* OR “cov” OR respiratory-syndrome OR “ncov” OR wuhan OR COVID*).kw,ti,ab.]

### Assessment of inclusion

Two authors (JB, NJ or IL) independently screened the retrieved titles and abstracts. Disagreements were resolved by discussion with other authors. The inclusion criteria were: (i) original research (not a review, guidelines, discussion, regulations, debate or commentary) published in English since January 1980; (ii) the research needed to describe face mask use that might prevent disease transmission or symptom development among people in the community (rather than prevent transmission to or from professionals in clinical settings); (iii) the study described an observed relationship between face mask use and respiratory symptoms or infection by respiratory pathogens: (e.g. influenza, coronavirus, tuberculosis); (iv) there was a contemporary comparator or control group (non-barrier users) for whom disease incidence data were also collected; (v) any study design in any country, as long as comparator data were available.

The full text of each article that passed screening was retrieved and eligibility verified as part of data extraction. 

### Data extraction for effectiveness

Characteristics of included studies, qualitative data and numbers of participants who developed respiratory outcomes in relevant study arms were extracted. The preferred specific outcome was influenza-like illness (ILI), defined by WHO as fever ≥ 38 C° with cough and onset ≤ 10 days before diagnosis [32]. Where a WHO-definition was unavailable, we accepted other similar case definitions (e.g. cold symptoms, acute respiratory infections, clinical cases of influenza or severe acute respiratory syndrome (SARS)) so that we could expand the evidence base and because of the often reported ‘atypical’ presentations and disease courses of COVID-19 [33]. Where studies reported three arms we extracted data for arms where the only difference was whether a face mask was worn (e.g. hand hygiene and no masks vs hand hygiene and face masks).

### Synthesis of evidence on effectiveness

Characteristics of included studies were tabulated. Numbers of suspected or confirmed infections and numbers of people at risk in each study arm were input to Review Manager 5.3 [34] for meta-analysis by JB, verified by other authors. We calculated pooled odds ratios (OR) using Mantel–Haenszel random effects meta-analysis (due to expected high heterogeneity) separately for primary prevention (when no cases were yet been identified) and prevention of secondary cases (when an individual was diagnosed with an infection and the aim was to prevent contacts from getting disease). We subgrouped by study design (RCT, cohort, case–control or cross-sectional), and presented these subgroups in forest plots without global pooling to understand consistency of evidence across study designs. We also showed the trend of evidence when outcomes were subgrouped by setting. For secondary transmission (in RCTs) we subgrouped by who wore the face mask: index case, well contacts (i.e. non-affected by the virus/respiratory illness in question) of the index case, or both. Outcomes after wearing face veils were also presented where evidence was available.

### Quality of evidence

Risk of bias of included RCTs was assessed (by LH) using the Cochrane risk of bias tool [35], and biases and limitations identified by primary study authors of observational studies were noted. We assessed the quality of evidence using the Grading of Recommendations, Assessment, Development and Evaluations (GRADE) framework. GRADE assessment was based on the RCT data and supported (strengthened) or contradicted (weakened) by observational data [35]. To further evaluate the translational value of the evidence, we report narratively on other aspects of the studies. Compliance or contamination (protocol violations) in RCTs were noted, along with any information about what kinds of masks controls wore as part of the contamination. Formal quality assessment checklists were not undertaken for observational studies, but we noted the kinds of masks worn (if reported). For all primary studies, settings and outcomes were recorded and are discussed with respect to their relevance to aspects of COVID-19 outbreak control. For all primary studies, we noted limitations as reported by the original investigators and discuss narratively any general limitations these imply for the wider evidence base.

### Ethical statement

Ethical approval was not required because this is an analysis of published aggregated secondary data that are not participant identifiable.

## Results

### Study selection and overview

**Figure 1:**
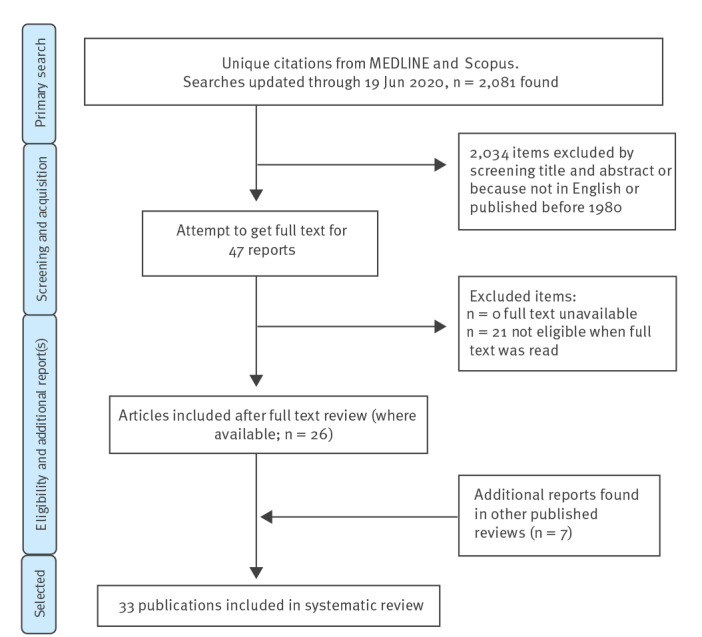
Study selection process of reports to review community use of face masks and similar barriers to prevent respiratory illness such as COVID-19, 1 January 1980–19 June 2020 (n = 2,081 studies)

Figure 1 shows the study selection process. The search was updated through 19 June 2020. Altogether, 1,233 titles and abstracts were retrieved from Scopus, and 1,657 from Embase with Medline. Our search located all 11 exemplar articles. Combining and de-duplicating left 2,081 articles. Of these, 236 were not written in English and 81 were published before 1980, so were removed. This left 1,764 titles and abstracts to screen, of which 47 were selected to be collected in full text. Full text review identified 26 eligible studies. Checking other systematic reviews on protective effects of face mask use in the community identified a further seven studies (five in the Hajj setting and two in other community settings). Among these total 33 eligible studies, the specific mask types were mostly unspecified, but where specified they were surgical medical grade items (n = 15).

COVID-19: coronavirus disease.

Study characteristics are shown in Table 1. Of the 33 included studies, 12 were designed as cluster-RCTs, five were cohort studies, six were case–control and 10 were cross-sectional. Data suitable for meta-analysis were reported in 31 studies. Settings included schools, university residences, visits to healthcare providers, family households, the Hajj mass gathering, and non-specific community places. Most studies reported on ILI as an outcome (n = 14) or respiratory illness (n = 10). Fever with respiratory symptoms, upper respiratory tract infection, laboratory-confirmed or clinical influenza, toxic pneumonitis, common colds, other respiratory symptoms, evidence of immunity to SARS-CoV-1 from serology and positive RT-PCR results for SARS-CoV-2 were also used as dichotomous outcomes when ILI was unavailable. All mass gathering studies were associated with the Hajj pilgrimage. Supplementary Table S1 lists additional characteristics of the included studies. GRADE assessments are shown in Table 2.

**Table 1:** Setting, study design and outcome for each included study in the review of community use of face masks and similar barriers to prevent respiratory illness, 1 January 1980–19 June 2020 (n = 33 studies)

Study	Setting	Design	Outcome	Comparison
Aiello 2010 pilot [21]	University residences	Cluster RCT	Respiratory illness	Allocated arms
Aiello 2012 [22]	University residences	Cluster RCT	ILI symptoms	Allocated arms
Alfelali 2019 as RCT [37]	Hajj pilgrimage	Cluster RCT	Respiratory illness	Allocated arms
Alfelali 2019 [37]	Hajj pilgrimage	As cohort	Respiratory illness	Used face mask daily or not
Al-Jasser 2012 [66]	Hajj pilgrimage	Cross sectional	Respiratory illness	Most of the time vs sometimes/never
Balaban 2012 [46]	Hajj pilgrimage	Retrospective cohort	Respiratory illness	Had face mask practice or not
Barasheed 2014 [67]	Hajj pilgrimage, pilgrims sleeping near index cases	Cluster RCT	Respiratory illness	Allocated arms
Canini 2010 [23]	Household with index case wearing mask who had been symptomatic < 48 hours	Cluster RCT	ILI	Allocated arms
Choudhry 2006 men [36]	Hajj pilgrimage (males)	Prospective cohort	Respiratory illness	Most of time vs sometimes/never
Choudhry 2006 women [36]	Hajj pilgrimage (female)	Prospective cohort	Respiratory illness	Most of the time vs sometimes/never
Cowling 2008 [25]	Household, wearing masks soon after index case influenza test	Cluster RCT	ILI	Allocated arms
Cowling 2009 [24]	Household, wearing masks soon after index case influenza test	Cluster RCT	ILI	Both arms also had hand hygiene intvn
Deris 2010 [48]	Hajj pilgrimage	Cross-sectional	ILI	Allocated arms
Emamian 2013 [68]	Hajj pilgrimage	Nested case–control	Respiratory illness (not colds)	Wore a mask or not
Fan 2020 [47]	Chinese citizens (82% students) living in Iran and subsequently evacuated	Cohort	Confirmed SARS-CoV-2	Wore a mask or not before left Iran
Hashim 2016 [41]	Hajj pilgrimage	Cross-sectional	Respiratory illness	Used or not; multiple types of face cover used
Jolie 1998 [69]	Pig farm, visiting students	Cross-sectional	Respiratory symptoms	During visit or not
Kim 2012 [70]	Schools	Cross-sectional	Laboratory-confirmed influenza	Continuous or irregular vs non-users
Larson 2010 [26]	Care settings	Cluster RCT	ILI	Allocated arms
Lau 2004a [28]	Public places, visitors	Case–control	ILI = suspected SARS	Frequently vs seldom/no
Lau 2004b [27]	Hospital, visitors to SARS index cases	Case–control	ILI = suspected SARS	During visit or not
MacIntyre 2009 [29]	Household, adults wear masks and care for sick child	Cluster RCT	ILI	Allocated arms
MacIntyre 2016 [44]	Household, index case wearing mask when symptomatic < 24 hours	Cluster RCT	ILI	Allocated arms
Shin 2018 control arm [71]	Community	Cohort	Common cold symptoms	Habitually wearing a face mask or not
Shin 2018 intvn arm [71]	Community	Cohort	Common cold symptoms	Habitually wearing a face mask or not
Simmerman 2011 [30]	Household	Cluster RCT	ILI	Allocated arms
Suess 2012 [31]	Household, members wearing masks when index case symptomatic < 48 hours	Cluster RCT	ILI	Allocated arms
Tahir 2019 [38]	Poultry farm, workers	Cross-sectional	Serological tests for A(H9N2) influenza	Always vs sometimes/never
Tuan 2007 [43]	Households with laboratory-confirmed SARS case	Cohort	SARS-CoV-1 positive serology	Sometimes/mostly vs never
Uchida 2017 [72]	Schools	Cross-sectional	Influenza	Mask wearing ever vs never
Wu 2004 [73]	Community	Case–control	SARS (WHO case definition)	Always vs sometimes/never
Wu 2016 [45]	Hospital, visitors without contact with known case	Cross-sectional	ILI	Habitually or not
Zein 2002 [39]	Hajj pilgrimage, masks supplied for all	Cross-sectional	URTI symptoms	Used masks or not
Zhang 2013a [74]	Long-haul flights	Case–control	ILI linked to H1N1 (WHO case definition)	Wore mask for entire flight or not
Zhang 2013b [42]	Households, self-quarantine with index patient	Case–control	Laboratory-confirmed influenza (H1N1)	Daily mask wearing or not

ILI: influenza-like illness; intvn: intervention; RCT: randomised controlled trial; SARS: severe acute respiratory syndrome; SARS-CoV-1: SARS coronavirus 1; SARS-CoV-2: SARS coronavirus 2; URTI: upper respiratory tract infection; WHO: World Health Organization.

**Table 2:** Masks compared with no masks for respiratory illness, summary of GRADE findings, review of community use of face masks and similar barriers to prevent respiratory illness, 1 January 1980–19 June 2020 (n = 33 studies)

Setting (outcome always ILI)	Study type	Anticipated absolute effects^a^ Risk expressed per 1,000^b^		Relative effect OR (95% CI)	Number of study participants (number of studies)	Quality of the evidence (GRADE)^c^	Comments
		Without masks	With masks (95% CI)				
Primary prevention, well wear masks	RCTs	108	102 (83–125)	0.94 (0.75–1.19)	5,183 (3 RCTs)	⨁⨁◯◯ LOW^d,e,f,g,h^	Wearing a mask may very slightly reduce the odds of primary infection with ILI by around 6^i^ to 15%^i^. Low-quality evidence (downgraded once each for risk of bias and imprecision).
	Cohort	197	173 (73–358)	0.85 (0.32–2.27)	5,217 (7 cohorts)		
	Case–control	405	210 (109–364)	0.39 (0.18–0.84)	1,501 (4 studies)		
	Cross-sectional	341	240 (189–306)	0.61 (0.45–0.85)	10,058 (8 studies)		
Secondary transmission, use of masks in homes, only ill person wears mask	RCTs	62	59 (34–102)	0.95 (0.53–1.72)	903 (2 RCTs)	⨁◯◯◯ VERY LOW^j,k^	When one household member becomes ill with an ILI the effect of their wearing a mask on the odds of house-mates developing ILI is unclear, as the evidence is of very low quality (downgraded once for risk of bias, twice for imprecision).
	Case–control	248	491 (328–657)	2.93(1.48–5.81)	162 (1 study)		
Secondary transmission, use of masks in homes, only well person(s) wear(s) mask(s)	RCTs	121	114 (86–150)	0.93 (0.68–1.28)	2,078 (2 RCTs)	⨁⨁◯◯ LOW^j,l^	House-mates wearing masks once another household member has contracted ILI may modestly reduce the odds of further household members becoming ill by around 7%. Low quality evidence (downgraded twice overall for risk of bias and imprecision).
	Cohort	45	47 (2–482)	1.04 (0.05–19.52)	163 (1 study)		
	Case–control	337	328 (203–486)	0.96 (0.50–1.86)	162 (1 study)		
Secondary transmission, use of masks in homes, both well and ill persons wear mask	RCT	120	100 (62–158)	0.81 (0.48–1.37)	1,605 (5 RCTs)	⨁⨁◯◯ LOW^l,m,n^	Both house-mates and the infected household member wearing masks once one household member has contracted ILI may modestly reduce the odds of further household members becoming ill by around 19%. Low quality evidence (downgraded twice overall for risk of bias, imprecision and inconsistency).
	Case–control	173	86 (36–188)	0.45 (0.18–1.10)	191 (1 study)		

CI: confidence interval; GRADE: Grading of Recommendations, Assessment, Development and Evaluations; ILI: influenza-like illness; OR: odds ratio; RCT: randomised control trial.

^a^ The risk in the intervention group (and its 95% CI) is based on the assumed risk in the comparison group and the relative effect of the intervention (and its 95% CI).

^b^ For each of the intervention (with mask) and comparison (without mask) groups, the risk is expressed as the number of group members who developed ILI or respiratory illness per 1,000 group members.

^c^ GRADE Working Group grades of evidence. HIGH quality: we are very confident that the true effect lies close to that of the estimate of the effect. MODERATE quality: we are moderately confident in the effect estimate; the true effect is likely to be close to the estimate of the effect, but there is a possibility that it is substantially different. LOW quality: our confidence in the effect estimate is limited; the true effect may be substantially different from the estimate of the effect. VERY LOW quality: we have very little confidence in the effect estimate; the true effect is likely to be substantially different from the estimate of effect.

^d^ Risk of bias: outcome assessors were not blinded for ILI (as outcomes are self-reported and participants could not be blinded), but were for laboratory-based diagnoses (not shown). Allocation concealment often unclear. Downgraded once.

^e^ Inconsistency: I^2^ was 19%. Evidence from other study designs were roughly confirmatory of a small beneficial effect. Not downgraded.

^f^ Indirectness: measured exactly what we wanted to know re primary prevention. Not downgraded.

^g^ Imprecision: the 95% CIs included both benefits and harms. Downgraded once.

^h^ Publication bias: no suggestion of publication bias, not downgraded.

^i^ The 6% comes from the OR of 0.94 (point estimate for RCTs), and the 15% comes from the OR of 0.85 (cohort studies). The RCTs and cohort studies are the two strongest study designs – the designs most likely to give us useful answers. As the RCTs probably underestimate effects, and cohorts overestimate effects the likely effect size is in the 95% CI below 0.94 (to 0.75) and in the 95% CI above 0.85 (to 2.27). The overlap of these areas is between ORs 0.94 and 0.85, or reductions of 6 to 15%.

^j^ Risk of bias: In most trials outcome assessors were not blinded (as outcomes are self-reported and participants could not be blinded), and allocation concealment was often unclear. Downgraded once.

^k^ Imprecision: the 95% CIs included both big benefits and big harms. Downgraded twice.

^l^ Imprecision: the 95% CIs included both benefits and harms. Downgraded once.

^m^ Risk of bias: In most trials outcome assessors were not blinded (as outcomes were self-reported and participants could not be blinded). Downgraded once in conjunction with inconsistency (footnote n).

^n^ Inconsistency: I^2^ was 53%. Downgraded in conjunction with risk of bias in footnote m (downgraded once between both factors).

The patient or population consisted of people without ILI, either in contact with a person with ILI (secondary transmission) or not (primary prevention). The setting included any setting. The intervention (or exposure) was advice to wear a mask and/or provision of masks (or wearing a mask). The comparison was no advice to wear a mask/advice to not wear masks (or not wearing a mask).

### Prevention of primary infection, subgrouping by study design

**Figure 2:**
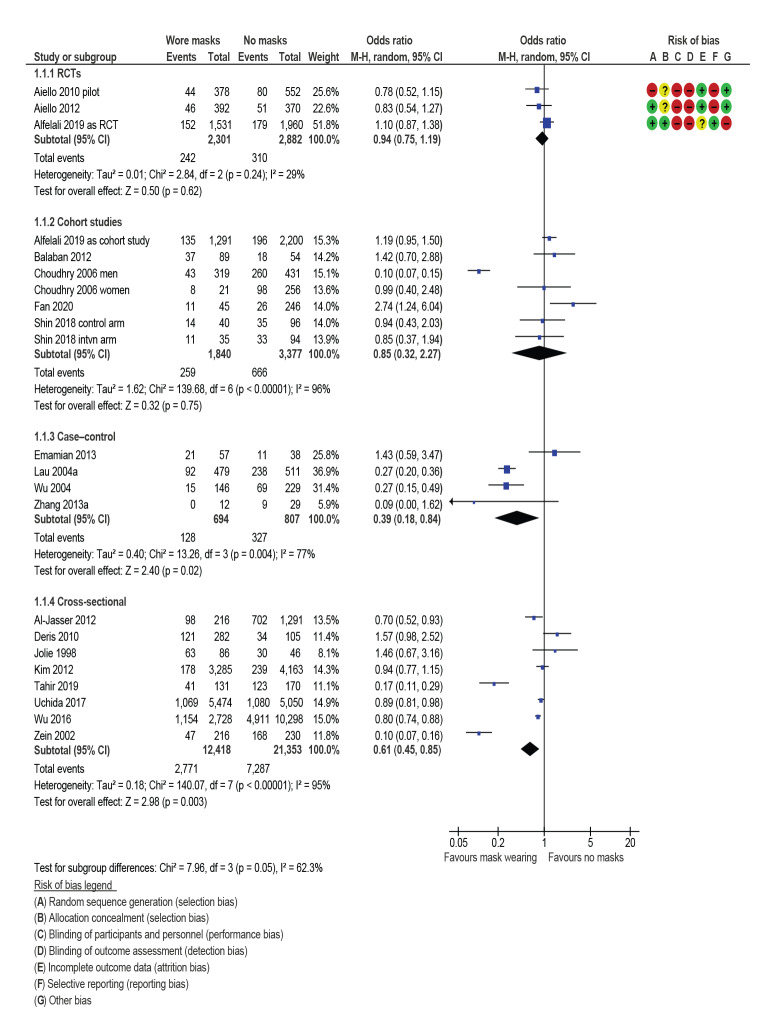
Mask wearing to prevent primary infection, by study design, review of community use of face masks and similar barriers to prevent respiratory illness, 1 January 1980–19 June 2020

CI: confidence interval; df: degrees of freedom; intvn: intervention; M-H: Mantel–Haenszel; RCT: randomised controlled trial.

Figure 2 shows grouping of results by study design. Pooled data are presented to calculate a single OR to compare and contrast study designs. Risk of biases for RCTs are also presented.

See Table 1 for study reference numbers, study setting, study design, outcome, comparison (when not allocated arms in RCTs) and any concurrent other intervention in both study arms.

The three RCTs, which measured the prevention of primary infection, indicated a slight, non-significant, reduction in the odds of primary infection with ILI (OR: 0.94; 95% CI: 0.75–1.19). Heterogeneity was low (I^2^ = 29%).

Evidence from the five cohort comparisons suggested face masks provided some primary protection (OR: 0.85; 95% CI: 0.32–2.27), although these findings were not significant. Heterogeneity was very high (I^2^ = 96%) and the men-only cohort from Choudhry et al. [36] was a noticeable outlier. This set of studies included observational data based on actual face-mask wearing habits from one study originally designed as an RCT [37].

Among four case–control (OR: 0.39; 95% CI: 0.18–0.84; I^2^ = 77%) and eight cross-sectional studies (OR: 0.61; 95% CI: 0.45–0.85; I^2^ = 95%), pooled data suggested that face-mask wearing was protective, but effects were highly heterogeneous. Of the cross-sectional studies, Tahir et al. [38] and Zein [39] were noticeable outliers. Removal of these outliers still indicates face-mask wearing as protective, although no longer significant, and heterogeneity falls slightly (OR: 0.89; 95% CI: 0.78–1.01; I^2^ = 64%, data not shown).

Two studies on primary prevention did not provide suitable data for pooling. Gautret et al. 2011 [40] gave no data but reported that they had done analysis supporting their conclusions to comment narratively that face masks were protective against respiratory tract infections. Another study without reported original data, Hashim et al. 2016 [41], concluded that respirators were not effective protection against ILI.

GRADE assessment suggested that wearing a mask may slightly reduce the odds of primary infection with ILI by around 6 to 15%. (i.e. somewhere between the effects seen in RCTs and the effects seen in cohort studies; likely to be the most robust of the observational studies). This was low-quality evidence (downgraded once each for risk of bias and imprecision) (Table 2).

### Prevention of primary infection by exposure setting

**Figure 3:**
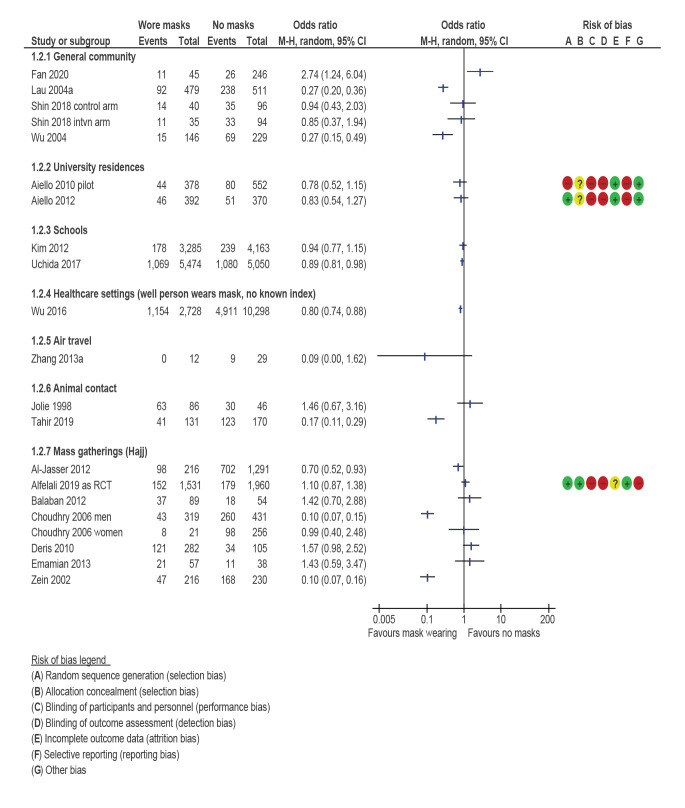
Mask wearing to prevent primary infection, by exposure setting, review of community use of face masks and similar barriers to prevent respiratory illness, 1 January 1980–19 June 2020

CI: confidence interval; intvn: intervention; M-H: Mantel–Haenszel; RCT: randomised controlled trial.

Figure 3 groups results by exposure setting. Pooling of data from different study designs is not appropriate to calculate a single OR statistic. Most results favoured face-mask wearing.

See Table 1 for study reference numbers, study setting, study design, outcome, comparison (when not allocated arms in RCTs) and any concurrent other intervention in both study arms.

Face-mask wearing was mostly protective (the midpoint-estimates of most included studies favoured face-mask wearing) in the general community (3 cohort and 2 case–control of which 2 studies were significantly protective), university residences (2 cluster-randomised RCTs, neither significant at p = 0.05) and in schools (2 cross-sectional studies, neither significantly protective).

One case–control study for visits to healthcare clinics without a known index patient suggested that mask wearing was significantly protective against primary infection. One case–control study on air travel suggested a protective but non-significant relationship between mask wearing and avoiding infection.

The results were less consistent (the point-estimates showed both protective and non-protective relationships) for animal contact (2 cross-sectional studies, 1 significant protective finding), and suggested masks were mostly not significant in getting or avoiding disease when used at mass gatherings (all Hajj pilgrims; 1 cluster-randomised RCT, 2 cohort, 1 case–control and 3 cross-sectional; 2 significant protective findings).

### Prevention of primary infection among face veil wearers

**Figure 4:**
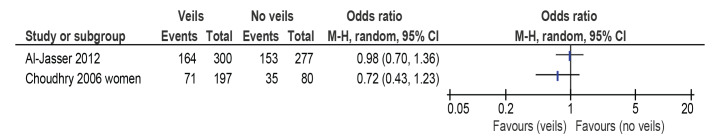
Face-veil wearing to prevent primary infection, review of community use of face masks and similar barriers to prevent respiratory illness, 1 January 1980–19 June 2020

CI: confidence interval; M-H: Mantel–Haenszel; RCT: randomised controlled trial.

Figure 4 shows data from two studies (cross-sectional and cohort) examining case incidence among women who wore face veils often/always while on Hajj pilgrimage. Both studies indicate a protective but non-significant relationship.

See Table 1 for study reference numbers, study setting, study design, outcome, comparison (when not allocated arms in RCTs) and any concurrent other intervention in both study arms.

### Secondary transmission

**Figure 5:**
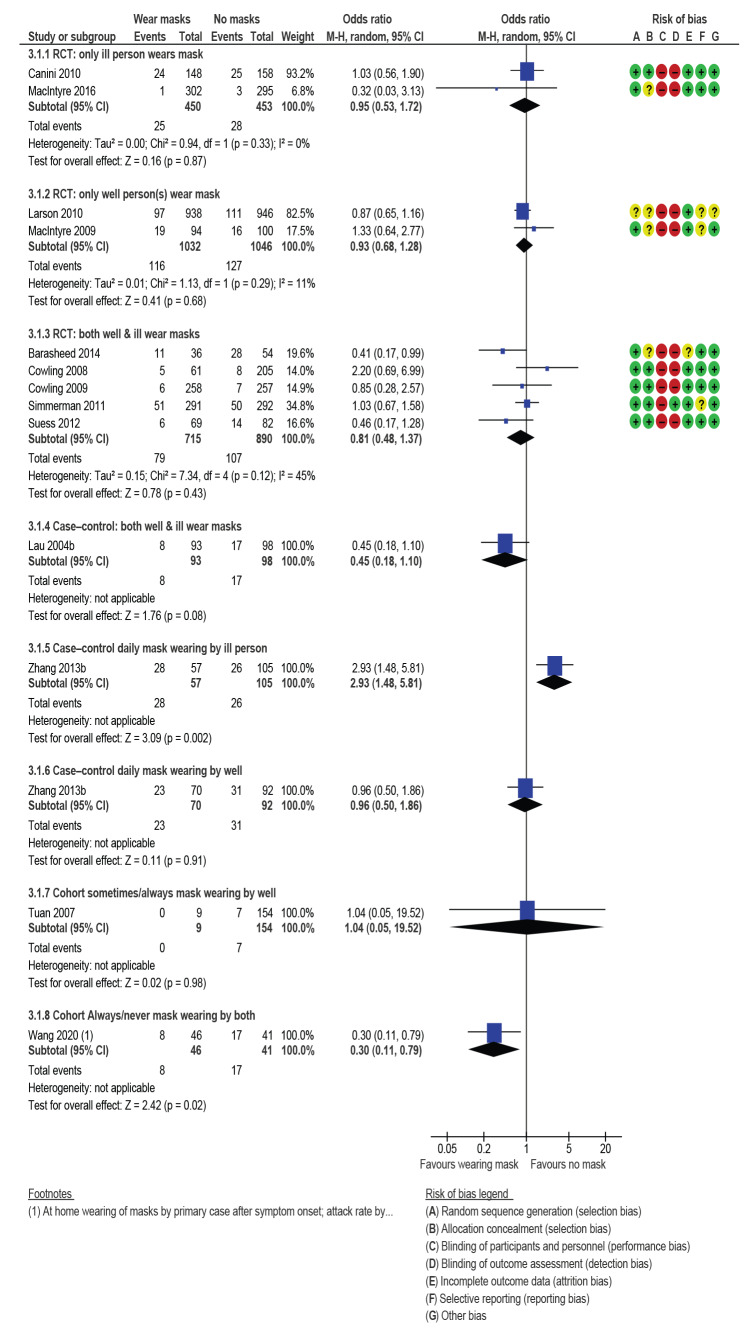
Mask wearing to prevent secondary infection, transmission mostly within households, review of community use of face masks and similar barriers to prevent respiratory illness, 1 January 1980–19 June 2020

CI: confidence interval; M-H: Mantel–Haenszel; RCT: randomised controlled trial.

Figure 5 shows results for secondary transmission subdivided by study design and who wore the face mask (index patient, well contacts or both). Presented are pooled data to calculate a single OR and risk of biases for each study design. Findings from the two RCTs when only infected persons wore a face mask, suggested a very small, non-significant protective effect (OR: 0.95; 95% CI: 0.53–1.72; I^2^ = 0%). The GRADE assessment suggested that the effect of the infected person wearing a face mask was unclear due to very low quality evidence (downgraded once for risk of bias, twice for imprecision).

See Table 1 for study reference numbers, study setting, study design, outcome, comparison (when not allocated arms in RCTs) and any concurrent other intervention in both study arms.

The protective effect was very small if only the well people wore face masks (OR: 0.93; 95% CI: 0.68–1.28; I^2^ = 11%; 2 RCTs). The GRADE assessment combining data from the two RCTs, and single cohort and case–control studies suggested low quality evidence. House-mates wearing masks once another household member has contracted ILI may modestly reduce the odds of further household members becoming ill by around 7%. Low quality evidence (downgraded twice overall for risk of bias, imprecision and inconsistency). 

Pooled data from five RCTs where both infected and non-infected household members wore face masks showed the odds of infection fell modestly and not significantly (OR: 0.81; 95% CI: 0.48–1.37; I^2^ = 45%).

Findings from the one case–control study (Lau 2004b on Figure 5) [27] where both infected and non-infected household members wore face masks indicated a large risk reduction but this was not significant at p < 0.05 (OR: 0.45; 95% CI: 0.18–1.10). Zhang et al. 2013b [42] is a case–control study that separated results for face-mask wearing by whether masks were worn by either index patient or contacts. These results significantly favoured no mask wearing by index patients (OR: 2.93; 95% CI: 1.48–5.81) and found negligible attack rate differences between case and control households when contacts wore masks (OR: 0.96; 95% CI: 0.50–1.86). The final comparison in Figure 5 draws data from a single cohort study [43] where 95% of contacts never wore masks during contact with confirmed SARS-CoV-1 cases. No significant effect from mask wearing (or not) was found (OR: 1.04; 95% CI: 0.05–19.52).

GRADE assessment for the five RCTs and the one case–control study suggested that both house-mates and the infected household member wearing masks once one household member has contracted ILI may modestly reduce the odds of further household members becoming ill by around 19%. This was low quality evidence (downgraded twice overall for risk of bias, imprecision and inconsistency).

### Secondary transmission and early commencement of face-mask wearing

**Figure 6:**
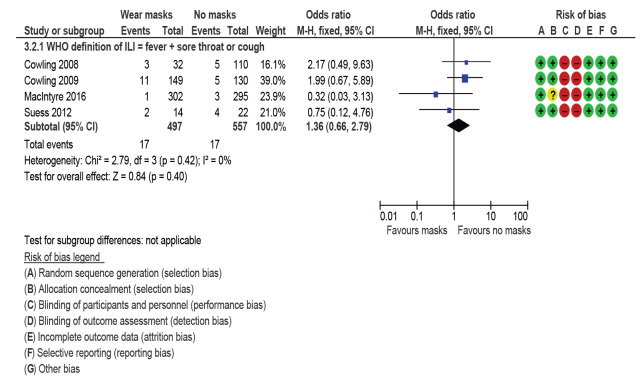
Mask wearing to prevent secondary infection starting < 36 hours after onset in index patient, transmission within households, review of community use of face masks and similar barriers to prevent respiratory illness, 1 January 1980–19 June 2020

CI: confidence interval; df: degrees of freedom; ILI: influenza-like illness; M-H: Mantel–Haenszel; RCT: randomised controlled trial; WHO: World Health Organization.

Figure 6 shows results for the four secondary transmission RCT studies providing data for attack rates when face-mask wearing started < 36 hours after index patient became symptomatic. The masks could be worn by either ill person, well person, or both (pooled comparison). A single OR statistic and risk of biases for RCTs are presented. Face-mask wearing was not protective in this subgroup analysis (OR: 1.36; 95% CI: 0.66–2.79; I^2^ = 0%). Some of the original investigators in these studies undertook logistic regression to adjust their findings for other confounders and found evidence that early face-mask wearing (< 36 hours after symptom onset) could be protective, but acknowledged that their models were underpowered.

See Table 1 for study reference numbers, study setting, study design, outcome, comparison (when not allocated arms in RCTs) and any concurrent other intervention in both study arms.

### Quality of evidence

Many of the included RCTs reported that participants did not follow instructions about wearing face masks [19,24,25,29,37,44]. Several reported that some controls wore face masks during the monitoring period [25,30,44], while many intervention participants did not wear face masks the majority of the time [24-26,29,44]. All of the RCTs included in our review provided specific face masks (usually surgical grade, rarely P2 or equivalent grade respirator) with instructions on how to wear the face masks, how often they should be changed and how to hygienically dispose of used face masks. No information was reported about the types of face masks that (contrary to protocol) some controls in RCTs used. Very few of the observational studies collected information about what type of face covering was used. Several studies highlight potential problems of recall bias [27,38,45]. Other studies note that potential confounding factors were not explored [42,46,47].

Apart from studies conducted during the Hajj, the evidence base for primary transmission in specific settings such as public transport, schools, cafeterias and shops was minimal (Figure 3). The only mass-gathering setting where face-mask wearing evidence has been gathered and published is the Hajj.

## Discussion

The quality of the evidence is problematic. We believe that RCT evidence underestimated efficacy while observational studies have overestimated how protective face-mask wearing can be because of unmeasured co-factors that cause confounding. For example, those who choose to wear masks may be more risk averse in general so undertake many protective activities alongside wearing a mask. Therefore, specific accurate estimates of the degree of protectiveness of face masks from the currently available evidence base are unreliable. Our best estimate is that the effect of wearing a face mask is between the effects seen in RCTs and the effects seen in cohort studies, or around 6 to 15% reduction in disease transmission.

Lack of evidence on transmission in specific settings is also problematic, given that effectiveness is likely to differ between settings, and infection control measures will need to vary by setting. The evidence is arguably insufficient to comment meaningfully on primary transmission reduction in any setting other than the Hajj. It is not ideal that the only mass gathering event studied is the Hajj which is exceptional for high contact rates over 10–20 days and which attracts a narrow demographic (older and relatively wealthy individuals) [39-41,48,49]. These features are unlike many other mass gatherings.

Producing clear evidence from observational and randomised studies that face masks are effective (or not) in slowing COVID-19 spread would be desirable. Only one of the studies included in this review were about people exposed to potential SARS-CoV-2 infection [47]. There has sometimes been resistance to wearing face coverings, recommended or mandated to try to slow spread of COVID-19 [50,51]. These tense conflicts seem likely to undermine public health measures intended to slow the spread of COVID-19. This situation underscores the need to produce reliable and clear primary research.

Population level studies that consider COVID-19 spread before and after mask-wearing policies (and combinations of other control measures) were introduced in various localities [52-56] have more often than not concluded that mask-wearing mandates or recommendations seemed to accelerate epidemic decline in early 2020. Analyses of impacts of non-pharmaceutical interventions (NPI) in the COVID-19 pandemic are preliminary and some have been criticised for indirect measurements, use of selective data and inappropriate analytical methods [57-59]. Compliance information is also not usually included in these natural experiment studies. It is not clear why population studies have tended to show definitive findings on mask wearing, which are not reflected in primary research. Aligning findings from the different evidence bases, and establishing a secure consensus about which NPI measures are effective, would be desirable and also might illuminate less recognised transmission pathways and best opportunities for risk reduction.

While RCTs may underestimate effects of face masks, because of compliance problems (contamination) in both intervention and control groups, compliance with mask wearing seems very likely to be partial in real life, too. This problem reflects a wider issue around public health interventions. Archie Cochrane himself pointed out “*the gulf, which has been much under-estimated, between the scientific measurements based on RCTs and the benefit measurement in the community*” [60]. There are in fact two questions here. The first is, do face masks, if used appropriately, reduce the risk of transmission from an infected individual and/or protect an uninfected person if in the presence of someone with COVID-19. The second question is whether public health interventions that require or encourage people to wear face coverings actually achieve their objective of reducing diseases in the wider population. Evidence is still emerging on this later and most important question.

### Limitations

Due to the rapidity of this review we did not consider other article archives or databases such as Google Scholar, CINAHL and medRXiv. Our search terms were designed to be more specific than they were sensitive. We addressed all types of respiratory symptoms and diagnoses; in reality, transmission pathways even among respiratory viruses do vary somewhat individually. A good reason to generate a larger evidence base is to make it possible to meaningfully separate pathogens and outcomes. ‘Mask’ had to be among title/abstract/keywords, and we are aware that ‘mask’ was more likely to be among the title/abstract/keywords if mask wearing was linked to significant effects. In practice, the search strategy meant that our search terms were slightly biased into finding articles where masks had been protective rather than having no effect. We also considered only dichotomous outcomes; we did not classify outcomes by severity of symptoms or other clinical outcomes [61]. It is possible that face-mask wearing reduced duration or severity of symptoms experienced due to reducing infectious dose received, although not actual disease.

We did not undertake cost–benefit analysis. The sudden emergence of COVID-19 led to high community demand for face barriers and raised valid concerns that insufficient supplies of face masks were available for healthcare workers [9,10]. The environmental and economic costs of regularly using face masks are notable, and only partly abated by reuse. Other efforts have been made to calculate the balance of all benefits and costs in face-mask wearing for disease prevention [62-65].

We make no comment on the relative utility of other proposed protective measures compared with face-mask wearing, such as self-isolation, distancing or frequent handwashing: we have not undertaken research on those measures for comparison. We did not formally assess likelihood of publication bias in the primary research evidence base. Only literature in English was reviewed, so we may have missed relevant reports in other languages.

### Conclusions

Original primary research is needed on whether and to what extent face masks reduce transmission of COVID-19 and other respiratory communicable diseases. Future RCT investigations should explore methods to enhance compliance in both intervention and control participants and ensure these are reported. All studies should report information about the types of face masks people wore (in both control and intervention arms), frequency of wear and (ideally) the range of other protective measures used. It would be helpful to understand how masks were used by research participants; e.g. if masks were washed, disinfected or how they were disposed of, as well as duration of wear. Future observational studies should carefully collect information on and adjust for key confounders. Research needs to be sensitive to settings and types of contact as well as the specific disease. The impact of when mask wearing starts and type of prevention (e.g. primary, early or later secondary prevention) needs investigating further, and is likely to differ between diseases. This is especially true if studies can be well powered to produce more definitive results, or if evidence should emerge about face mask use within homes before symptom onset or within a very short period (perhaps 4–12 hours) after symptom onset.

## Supplementary Data

238000 Supplementary Material
