# Supplementary material for: Community use of face masks and similar barriers to prevent respiratory illness such as COVID-19: a rapid scoping review
Source: Euro Surveill. 2020 Dec 10;25(49):2000725. doi: 10.2807/1560-7917.ES.2020.25.49.2000725 (PMC7730486; doi:10.2807/1560-7917.ES.2020.25.49.2000725)
Supplement: Supplementary Material [file 2000725_Supplement.pdf]

This supplementary material is hosted by Eurosurveillance as supporting information alongside the article **Community use of facemasks and similar barriers to prevent respiratory illness such as COVID-19: A rapid scoping review**, on behalf of the authors, who remain responsible for the accuracy and appropriateness of the content. The same standards for ethics, copyright, attributions and permissions as for the article apply. Supplements are not edited by Eurosurveillance and the journal is not responsible for the maintenance of any links or email addresses provided therein.

Supplementary Table S1 Included Study Characteristics

Study name and design	Who wore masks (or not)	Setting	When potentially exposed; Duration of monitoring	Outcomes reported	Type of face barrier	Author conclusions	Limitations or possible biases stated by study authors
**Primary prevention, Community settings not mass gatherings**							
Aiello et al 2010 [1]<br><br>Cluster RCT	Well university students	University of Michigan halls of residence	2006-2007 flu season: 26 Jan-16 March 2007<br><br>6 weeks	ILI AND influenza	Medical masks with ear loops (TECNOL procedure masks; Kimberly-Clark)	Mask-wearing led to reduction in ILI in weeks 4-6	Trial interrupted by spring break, in weeks 4-5 of monitoring. Mask-only group wore masks 3.92 (sd 3.31) hrs/day.
Aiello et al. 2012 [2, 3]<br><br>Cluster RCT	Well university students	University of Michigan halls of residence	2007-2008 flu season<br><br>6 weeks	ILI AND influenza	Medical masks with ear loops (TECNOL procedure masks; Kimberly-Clark)	Mask-wearing led to reduction in ILI in weeks 3-6	Adherence to wearing masks was variable; students only wore masks within halls, average 5.04 (sd 2.2) hrs/day. Transmission was quite possible outside halls; reliance on self-reported data. Average 11.86% of observation shifts that any mask was seen worn correctly.
Fan et al 2020 [4]<br><br>Cohort	Chinese evacuees, all ages but 82% were university students	Iran, community dwelling	2020	Swab test, PCR confirmed SARS-CoV-2	"facemask"	wearing a facemask while in Iran increased the risk for COVID-19 infection (χ^2 = 7.902, p = 0.005)	Did not estimate spatial risk factors affecting SARS-CoV-2 infection in Iran and potential risk in China because of the relatively small number of cases and the short study period.
Jolie et al 1998 [5]<br><br>X-sectional	Veterinary students	USA WI (south central) pig farm	exposed Oct 1993/Oct 1994<br><br>single surveys post farm visit (unclear how long after)	Toxic pneumonitis	Preformed cone style facemask (Deseret Filtermask cat. no. 375201)	The presence of respiratory and/or general symptoms was not significantly different between mask users and non-users.	Not as rigorous as an RCT, single strap mask design may have had an impact.
Kim et al 2012 [6]<br><br>X-sectional	Children age 7-18 yrs	South Korea school pupils	18 Nov-8 Dec 2009 monitoring	H1N1 flu	"facemask"	Continuous use of facemasks exhibited a significant negative relationship with H1N1 infection (OR 0.51, 95% CI 0.3-0.88)	This study was testing an hypothesis that obesity would affect influenza risk, was not designed to assess masks as protective/risk factors. Reliant on self-reporting by children.
Lau et al 2004a [7]<br><br>Case-control	Community dwellers	Within and outside of Hong Kong HHs	spring 2003	ILI = likely SARS	"facemask"	Masks were protective 0.36 (0.25-0.52)	Respondents may have interpreted questions incorrectly and inconsistently, including what wearing a mask "frequently" vs. "occasionally" meant.

Study name and design	Who wore masks (or not)	Setting	When potentially exposed; Duration of monitoring	Outcomes reported	Type of face barrier	Author conclusions	Limitations or possible biases stated by study authors
Shin et al 2018 [8]<br><br>Observational data collected during an RCT	healthy adult volunteers age 20-65	Community dwelling near Kyushu University, Japan	Jan-March 2015<br><br>8 weeks	Common cold	"facemasks"	Habit of wearing facemasks made no difference	Not commented upon with respect to masks; trial was designed to test supplements that might reduce or prevent common cold. Facemask 'habit' was not defined.
Tahir et al 2019 [9]<br><br>X-sectional	People in jobs with contact with poultry (wide range of jobs)	Rawalpindi, Pakistan	Surveyed and sampled December 2016-May 2017	Seroprevalence for antibodies against H9 influenza	"facemasks"	Persons who used facemasks had significantly lower (P < 0.002) seroprevalence (29.6%) than those who never used them (90.6%) Facemask used: sometimes/never/always positive serological result: 64%/91%/31%; p=0.001	Insufficient distinction for type of contact/poultry farming; x-sectional design meant they missed seasonal trends. Reliant on self-report; always/sometimes/never durations not clearly defined.
Uchida et al 2017 [10, 11]<br><br>X-sectional	Healthy primary school children (age 7-12yrs)	Matsumoto City, Nagano, Japan.	2014/2015 flu season<br><br>surveyed end Feb 2015 about most recent winter period	Influenza	"facemask"	The overall effectiveness of … mask wearing was … 8.6%... wearing masks (0.859, 0.778–0.949) had significant protective association	Expected they had missed sub-clinical and asymptomatic cases; Limited age sample; x-sectional rather than RCT design. Mask wearing was simple yes/no, self-report by children's guardians. No indication of frequency of use.
Wu et al 2004 [12]<br><br>Matched case-control	Cases and matched controls (in community, lacked known contact)	Beijing PRC	28 April to 16 Jun 2003	ILI = likely SARS (clinical illness definition)	"facemask" when going out	Wearing masks outside the home was strongly protective; more protection the more they reported wearing the masks. wore a mask never =ref, sometimes OR 0.5 (0.2 to 0.9) p=0.02 always OR 0.3 (0.2 to 0.6) p= <0.001	Self-report, self-selection in those who answered; uncertainty if SARS; some refusal of blood tests; likely over diagnosis.
Wu et al 2016 [13]<br><br>X-sectional	Community dwelling people going to hospitals for any purpose	Beijing PRC	January 2011 survey<br><br>About illness in previous year	ILI	"facemask"	Habit of facemask use when going to hospitals (OR 0.87; 95% CI 0.80–0.95) was protective.	Recall bias; ILI symptoms from non-pathogen cause(s); no pandemic at time led to case number.
Zhang et al 2013a [14]<br><br>case-control	Airplane passengers	Flights between to Hong Kong, Vancouver and New York NY	Three long haul flights, May 2009	Influenza A(H1N1)pdm09; ILI	Unknown, authors stated "presumably, individually acquired masks represented a mixture of N95 and other less efficient masks"	None of the 9 case-passengers, compared with 47% (15/32) of control passengers, wore a facemask for the entire flight (odds ratio 0, 95% CI 0–0.71).	Unclear what type of mask people used, lack of seating information, severity of illness information, missing case passengers, lost many to follow-up (limited sampling frame), recommended an RCT. Not clear if passengers wore mask entire or how much of flight duration.
**Household case-control studies, secondary attacks**							

Study name and design	Who wore masks (or not)	Setting	When potentially exposed; Duration of monitoring	Outcomes reported	Type of face barrier	Author conclusions	Limitations or possible biases stated by study authors
Lau et al 2004b [15]<br><br>Case-control	index pt &/or contracts of known pts (has these as separate RFs); visit at home or in hospital	Hong Kong	spring 2003	ILI = likely SARS	"facemask"	Wearing masks to visit index patients in hospital/patient's separate home reduced transmission (Sec Attk rates): n=2195 (table 6) OR 3.12 (1.65 to 5.91) both without mask table 6 and OR 4.16 (2.37 to 7.30) both without mask, table 1	Known patient may not be source of infection in others; other nosocomial infection possible; case definition was nonspecific; interviewee sometimes was not a case but someone else in same HH, recall bias. Unclear if masks were worn for full duration of visit.
Zhang et al 2013b[16]<br><br>Case-control	Either index or contacts (no separate results for both)	Beijing China HHs subject to quarantine	August 1 - September 30, 2009	RT-PCR confirmed H1N1 influenza	"masks"	Cases were more common HHs where index wore masks (51% vs. 24% in control HHs). No significant differences between case and control groups, or whether index patients or contacts wore a mask daily. Hand-washing >3x/day was protective, however.	Recall bias, no ascertainment of asymptomatic infections, participants were an unusual subset of self-quarantined population.
**Household cohort studies, secondary attack**							
Tuan et al 2007 [17]<br><br>Cohort	Well persons who had close contact with index	Vietnam HH and community contacts	Retrospective survey using serology	Serological evidence (ELISA) of SARS-CoV infection in contacts	"mask"	Ninety-five percent of contacts reported never wearing a mask during contact with the SARS case.	Authors did not comment on mask wearing as a risk factor. Few people (5%, n=9) had worn masks. At least some of those 9 wore masks only 'sometimes'.
**Household (cluster) RCTs to prevent secondary attacks**							
Canini et al 2010 [18]<br><br>Cluster RCT	Sick person who tested + for flu < 48hrs after symptom onset, in HH with others	Within French HHs	2008-2009 flu season<br><br>Surveyed 7 days after recruitment	Influenza, ILI	Surgery masks with earloops, 3 plys, anti fog (AEROKYNH, LCH medical products)	Mask wearing was not protective	Too underpowered to make conclusions, no laboratory confirmation, discomfort reported with mask use. Average count of masks used was about 60% of expected use over period.
Cowling et al 2008 [19]<br><br>Cluster RCT	HHs where someone tested + for flu, started wearing < 36 hrs after +test	Hong Kong HHs	Feb-Sept 2007 exposure period<br><br>9 days after start wearing masks	Influenza; ILI	Tecnol – The Lite One, Kimberly Clark, Roswell, GA)	Neither masks nor handwashing intervention were protective in general, but subgroup for those who put on masks when index patient < 36 hours symptomatic suggested masks were protective	Attrition, bias in recruitment. Compliance not high and contamination noted: eg., >25% of contacts did not wear a mask at all, 30% of index cases in the control group wore masks at home; underpowered
Cowling et al 2009 [20]<br><br>Cluster RCT	HHs where someone tested + for flu, starting	Hong Kong HHs	2 Jan - 30 Sept 2008<br><br>7 days after start wearing masks	Influenza; ILI	Tecnol – The Lite One, Kimberly Clark, Roswell, GA)	Hand hygiene and facemasks can reduce influenza virus transmission if implemented early after symptom onset in an index patient; else intervention wasn't effective	Delay in donning masks after symptom onset may be too large; recruitment bias. 5% of contacts wore masks, 31% of index patients wore masks, 26% of intervention

Study name and design	Who wore masks (or not)	Setting	When potentially exposed; Duration of monitoring	Outcomes reported	Type of face barrier	Author conclusions	Limitations or possible biases stated by study authors
	soon after +test						contacts wore masks often/always; 49% of intervention index patients worse masks as directed.
Larson et al. 2010 [21]<br><br>Cluster RCT	Mostly care givers in close proximity, also ill patients	Manhatten HHs	2007-2009<br><br>Monitoring over 19 months	ILI	Procedure Facemasks for adults and children, Kimberly-Clark	[Although] compliance with mask wearing was poor, mask wearing [was] associated with significantly lower secondary transmission rates (p = 0.02).	Compliance was described as low, both for mask-wearing and symptom reporting.
MacIntyre et al 2009 [22]<br><br>Cluster RCT	all (min2) adults caring for index child = 0-15 yrs; at all times around child	Within Sydney Australia HHs	Aug-Oct 2006 & Jun-Oct 2007<br><br>7 days after masks issued	Respirat'y illness = ILI = fever &/or other symptoms	3M surgical mask, cat. no. 1820; *and* 3M flat-fold P2 mask, cat. no. 9320	Neither mask was effective	No fit-testing for P2 masks. Possible some patients were incubating when recruited; prospective study would be better. Between 31% and 38% of intervention group wore masks (of whole group supposed to wear masks).
MacIntyre et al 2016 [23]<br><br>Cluster RCT	Only index cases (unwell people)	Beijing PRC, within HHs	18 Nov-20 Jan recruited people who had symptoms < 24 hrs and been to hospital; no other ILI history in HH in previous 14 d<br><br>7 days after recruited	ILI: clinical respirat'y illness (list of symptoms stated); also lab Test detected virus infections	3M 1817 surgical mask	Rates of clinical respiratory illness (relative risk (RR) 0.61, 95% CI 0.18 to 2.13), ILI (RR 0.32, 95% CI 0.03 to 3.13) and laboratory-confirmed viral infections (RR 0.97, 95% CI 0.06 to 15.54) were consistently lower in the mask arm compared with control, although not statistically significant. A post hoc comparison between the mask versus no-mask groups showed a protective effect against clinical respiratory illness HazRat = 0.22 (0.06 to 0.86), but not against ILI and lab-confirmed viral respiratory infections.	Underpowered; participants removed masks to eat together. There was contamination = average 1.4 hrs/day mask use in controls compared to average wearing = 4.4 hrs/day in intervention. 35% of control index patients wore masks. 7/123 index patients in intervention arm didn't wear mask at all.
Simmerman et al 2011 [24]<br><br>Cluster RCT	index ill child & all healthy HH members	near Bangkok, Thailand; index pts = kids age 1m-15yrs	Recruited 9 Apr-13 Aug 2009<br><br>3 weeks after start mask	Influenza; ILI	50 standard paper surgical facemasks and 20 pediatric facemasks (Med-con co. Thailand #14IN-20AMB-30IN)	Influenza transmission [& ILI] was not reduced by interventions to promote hand washing and facemask use.	Study was complicated by arrival of 2009 H1N1 influenza pandemic and subsequent national hygiene campaign that may have changed behaviour in control group (possible contamination). 23% of control index patients wore masks, 90% of them after June 2009.

Study name and design	Who wore masks (or not)	Setting	When potentially exposed; Duration of monitoring	Outcomes reported	Type of face barrier	Author conclusions	Limitations or possible biases stated by study authors
Suess et al 2012 [25, 26]<br><br>Cluster RCT	both index patient & HH contacts of index pt, starting <2d after symptom onset	Berlin, Germany households	2009-2011<br><br>8 days after start wearing masks	Influenza; ILI	Child's Facemask, Kimberly-Clark, USA, or adults: Aérokyn Masques, LCH Medical Products, France	When analysing only households where intervention was implemented within 36 h after symptom onset of the index case, secondary infection in the pooled M and MH groups was significantly lower compared to the control group (adjusted odds ratio 0.16, 95% CI, 0.03-0.92). In a per-protocol analysis OR were significantly reduced among participants of the M group (adjusted odds ratio, 0.30, 95% CI, 0.10-0.94). Also reported lower sec-atk-rate the less they were at home.	Cannot say who wearing the mask reduced transmission (patient or contacts); There was limited testing for same influenza subtype that index patient had; variable implementation protocols. There was mask-wearing in 2/30 control households, while 55% of intervention households reported wearing masks often/always.
**Hajj pilgrims (mass gatherings)**							
Alfelali et al 2019 [27]<br><br>Cluster RCT but we also treated data as observation in meta-analysis due to contamination	Well pilgrims age 18+	Hajj pilgrimage events	2013–2015 (late Sept-mid Oct)<br><br>5 days during Hajj	Viral respirat'y infection	3MTM Standard Tie-On surgical mask, Cat No: 1816	Facemask did not prevent clinical or laboratory-confirmed viral respiratory infections among most Hajj pilgrims, although some subgroup differences were found, and clinical respiratory infections were lower in the intervention arm (13% versus 10%, p=0·03)	Short duration (4-5 days) of follow-up. Only 25% of intervention participants used facemasks daily, while 14% of control participants used masks daily. Controls were allowed to wear masks if they wanted
Al-Jasser et al. 2012 [28]<br><br>X-sectional survey	Pilgrims	Hajj pilgrimage events	Nov-Dec 2009<br><br>slightly variable, within two weeks of finish	Upper respirat'y tract infections	"facemasks"	Increased protection with increased habit of wearing masks. Unadj. model results: Most of time worn = referent, Sometimes worn had RR 1.17 (1.0-1.38) & Never had RR 1.21 (1.06-1.43) p = 0.14, but no effect in adjusted models.	Self reported outcome, self-selected participants, recall bias, lack of demographic data on respondents
Balaban et al 2012 [29]<br><br>pre-post travel surveys	Pilgrims	Hajj pilgrimage events	2009 Hajj (Nov-Dec)<br><br>Implies most were on Hajj for 2-4 weeks	Respirat'y Illness; ILI	"facemasks"	Not possible to determine the effectiveness of facemasks from these data, although wearing masks was associated with greater likelihood of illness (not statistically significant)	Problems with generalisability, no evaluation of pre-Hajj health information, likely under-ascertainment, self reported adherence without independent collaboration
Barasheed et al 2014 [30]<br><br>Cluster RCT	Pilgrims age 15+ in tents with a person ill ≤ 3d, both ill & any well who slept in adjacent bed to wear masks	Hajj pilgrims Mina Valley, Mecca, Saudi	early November 2011<br><br>5 days during Hajj, after recruitment	ILI; respirat'y symptoms	Surgical facemasks = (3M™ Standard Tie-On Surgical Mask, Cat No: 1816	More symptoms in the no-mask group, but laboratory test for pathogens found no difference between groups	Small number of participants. 56/75 (76% of intervention group were deemed compliant); 12% of controls (11/89) wore masks.

Study name and design	Who wore masks (or not)	Setting	When potentially exposed; Duration of monitoring	Outcomes reported	Type of face barrier	Author conclusions	Limitations or possible biases stated by study authors
Choudhry et al 2006 [31]<br><br>Prospective cohort	Unsymptomatic Saudi Pilgrims from Riyadh at recruitment; separated results for males and females	Hajj activities	2002 Hajj (core dates 20-25 Feb)<br><br>Surveyed about 2 weeks after recruitment	Acute respirat'y symptoms	mostly masks, also veils	Strongly increased risk of having ARI the less they used the mask (men)	Using the facecover as a proxy for a facemask in women may be leading to misclassification of exposure status.<br><br>Men, however, were using the facemask as a personal hygiene measure, independent of the place where they were, so suggested separate data by sex
Deris et al 2010 [32]<br><br>X-sectional survey	Malaysian pilgrims	Hajj pilgrimage events	2007 Hajj (17-22 Dec 2007)<br><br>Questions covered entire Hajj pilgrimage period	Respirat'y symptoms ; ILI	Unclear type of facemask, but implies not respirators	Wearing facemasks was associated with more symptoms, but not statistically significant, and no real controls	Very few unaffected persons: only 3.6% did not have at least one respiratory symptom
Emamian et al 2013 [33]<br><br>Prospective Nested case-control study	Iranian pilgrims	Hajj pilgrimage events	November 2010<br><br>Monitoring was simultaneous	Respirat'y tract infections that were not colds	"facemasks"	Masks not even effective in univariate	Small sample size
Gautret et al 2011 [34]<br>X-sectional survey	French pilgrims	Hajj pilgrimage events	November 2009 core dates, asked about entire visit, usually 4 weeks	Acute respirat'y symptoms	Surgical facemasks	Influenza vaccine and facemask use did not significantly reduce respiratory symptoms.	Low incidence may have been due to under reporting
Hashim et al 2016 [35]<br><br>X-sectional survey	Malaysian pilgrims	Hajj pilgrimage events	2013 Hajj (12-17 October are core dates); most had 2-4 week stay	Respirat'y illness; ILI	Distinguished: N95, surgical, wet or dry towel and veil	Facemask wearing not signif in unadjusted or adj model; 78% had ILI & 93.4% had respy symptoms, even though about 84% said they used masks	Inadequate sample size, poor response rate, loss to followup from # originally recruited.<br>(no data suitable for pooling)
Zein 2002 [36]<br><br>X-sectional	pilgrims	Hajj pilgrimage events	2002, 20-25 Feb are core dates<br><br>Unclear if symptoms collected from daily diaries or in single survey afterwards	Acute upper respirat'y tract infections	Maybe nose+mouth cotton masks were supplied by Indonesian Hajj committee	Masks were very protective<br>No-mask user OR = 3.10 (Confidence limit : 2.65 < MHRR < 3.82 ; p < 0.000).	Other covariates such as humidity or dust level not adjusted for

Note to Table 1: HH = Household(s)

References

1. Aiello AE, Murray GF, Perez V, et al. Mask use, hand hygiene, and seasonal influenza-like illness among young adults: A randomized intervention trial. *Journal of Infectious Diseases* 2010;201(4):491-98. doi: 10.1086/650396
2. Aiello AE, Coulborn RM, Perez V, et al. A randomized intervention trial of mask use and hand hygiene to reduce seasonal influenza-like illness and influenza infections among young adults in a university setting: International Journal of Infectious Diseases. Conference: 14th International Congress on Infectious Diseases (ICID). Miami, FL United States. Sponsor: Pfizer, Sanofi-Pasteur, American Society for Microbiology (ASM), Associacion Panamericana de Infectologia (API), Astellas Pharma Global Development, Inc. . Conference Publication: (var.pagings). 14 (SUPPL. 1) (pp e320), 2010. Date of Publication: March 2010. 2010.
3. Aiello AE, Perez V, Coulborn RM, et al. Facemasks, hand hygiene, and influenza among young adults: A randomized intervention trial. *PLoS ONE* 2012;7(1):e29744.
4. Fan J, Liu X, Shao G, et al. The epidemiology of reverse transmission of COVID-19 in Gansu Province, China. *Travel Medicine and Infectious Disease* 2020:101741.
5. Jolie R, Backstrom L, Thomas C. Health problems in veterinary students after visiting a commercial swine farm: Canadian journal of veterinary research = Revue canadienne de recherche veterinaire. 62 (1) (pp 44-48), 1998. Date of Publication: Jan 1998. 1998.
6. Kim CO, Nam CM, Lee DC, et al. Is abdominal obesity associated with the 2009 influenza A (H1N1) pandemic in Korean school-aged children? *Influenza and other Respiratory Viruses* 2012;6(5):313-17. doi: 10.1111/j.1750-2659.2011.00318.x
7. Lau JTF, Tsui H, Lau M, et al. SARS Transmission, Risk Factors, and Prevention in Hong Kong: Emerging Infectious Diseases. 10 (4) (pp 587-592), 2004. Date of Publication: April 2004. 2004.
8. Shin K, Wakabayashi H, Sugita C, et al. Effects of orally administered lactoferrin and lactoperoxidase on symptoms of the common cold. *International Journal of Health Sciences* 2018;12(5):44-50.
9. Tahir MF, Abbas MA, Ghafoor T, et al. Seroprevalence and risk factors of avian influenza H9 virus among poultry professionals in Rawalpindi, Pakistan. *Journal of Infection and Public Health* 2019;12(4):482-85. doi: 10.1016/j.jiph.2018.11.009
10. Uchida M, Kaneko M, Hidaka Y, et al. Effectiveness of vaccination and wearing masks on seasonal influenza in Matsumoto City, Japan, in the 2014/2015 season: An observational study among all elementary schoolchildren. *Preventive Medicine Reports* 2017;5:86-91. doi: 10.1016/j.pmedr.2016.12.002
11. Uchida M, Kaneko M, Hidaka Y, et al. High vaccination coverage is associated with low epidemic level of seasonal influenza in elementary schools: An observational study in Matsumoto City, Japan: BMC Infectious Diseases. 18 (1) (no pagination), 2018. Article Number: 128. Date of Publication: 13 Mar 2018. 2018.
12. Wu J, Xu F, Zhou W, et al. Risk Factors for SARS among Persons without Known Contact with SARS Patients, Beijing, China. *Emerging Infectious Diseases* 2004;10(2):210-16. doi: 10.3201/eid1002.030730
13. Wu S, Ma C, Yang Z, et al. Hygiene behaviors associated with influenza-like illness among adults in Beijing, China: A large, population-based survey: PLoS ONE. 11 (2) (no pagination), 2016. Article Number: e0148448. Date of Publication: 01 Feb 2016. 2016.
14. Zhang L, Peng Z, Ou J, et al. Protection by face masks against influenza A(H1N1)pdm09 virus on trans-pacific passenger aircraft, 2009. *Emerging Infectious Diseases* 2013;19(9):1403-10. doi: 10.3201/eid1909.121765

15. Lau JTF, Lau M, Kim JH, et al. Probable Secondary Infections in Households of SARS Patients in Hong Kong: Emerging Infectious Diseases. 10 (2) (pp 235-243), 2004. Date of Publication: February 2004. 2004.
16. Zhang D, Liu W, Yang P, et al. Factors associated with household transmission of pandemic (H1N1) 2009 among self-quarantined patients in Beijing, China. *PloS one* 2013;8(10)
17. Tuan P, Horby P, Dinh P, et al. SARS transmission in Vietnam outside of the health-care setting. *Epidemiology & Infection* 2007;135(3):392-401.
18. Canini L, Andreoletti L, Ferrari P, et al. Surgical mask to prevent influenza transmission in households: A cluster randomized trial. *PLoS One* 2010;5(1)
19. Cowling BJ, Fung ROP, Cheng CKY, et al. Preliminary findings of a randomized trial of non-pharmaceutical interventions to prevent influenza transmission in households. *PLoS ONE* 2008;3(5):e2101.
20. Cowling BJ, Chan KH, Fang VJ, et al. Facemasks and hand hygiene to prevent influenza transmission in households: A cluster randomized trial. *Annals of Internal Medicine* 2009;151(7):437-46. doi: 10.7326/0003-4819-151-7-200910060-00142
21. Larson EL, Ferng YH, Wong-McLoughlin J, et al. Impact of non-pharmaceutical interventions on URIs and influenza in crowded, urban households. *Public Health Reports* 2010;125(2):178-91. doi: 10.1177/003335491012500206
22. Maclntyre CR, Cauchemez S, Dwyer DE, et al. Face mask use and control of respiratory virus transmission in households. *Emerging Infectious Diseases* 2009;15(2):233-41. doi: 10.3201/eid1502.081167
23. MacIntyre CR, Zhang Y, Chughtai AA, et al. Cluster randomised controlled trial to examine medical mask use as source control for people with respiratory illness. *BMJ Open* 2016;6(12) doi: 10.1136/bmjopen-2016-012330
24. Simmerman JM, Suntarattiwong P, Levy J, et al. Findings from a household randomized controlled trial of hand washing and face masks to reduce influenza transmission in Bangkok, Thailand. *Influenza and other Respiratory Viruses* 2011;5(4):256-67. doi: 10.1111/j.1750-2659.2011.00205.x
25. Suess T, Remschmidt C, Schink S, et al. Facemasks and intensified hand hygiene in a German household trial during the 2009/2010 influenza A(H1N1) pandemic: Adherence and tolerability in children and adults. *Epidemiology and Infection* 2011;139(12):1895-901. doi: 10.1017/S0950268810003006
26. Suess T, Remschmidt C, Schink SB, et al. The role of facemasks and hand hygiene in the prevention of influenza transmission in households: Results from a cluster randomised trial; Berlin, Germany, 2009-2011. *BMC Infectious Diseases* 2012;12 doi: 10.1186/1471-2334-12-26
27. Alfelali M, Haworth EA, Barasheed O, et al. Facemask versus No Facemask in Preventing Viral Respiratory Infections During Hajj: A Cluster Randomised Open Label Trial. *SSRN (Lancet preprints)* 2019
28. Al-Jasser FS, Kabbash IA, AlMazroa MA, et al. Patterns of diseases and preventive measures among domestic hajjis from Central, Saudi Arabia. *Saudi Med J* 2012;33(8):879-86.
29. Balaban V, Stauffer WM, Hammad A, et al. Protective practices and respiratory illness among US travelers to the 2009 Hajj. *Journal of Travel Medicine* 2012;19(3):163-68.
30. Barasheed O, Almasri N, Badahdah AM, et al. Pilot randomised controlled trial to test effectiveness of facemasks in preventing influenza-like illness transmission among Australian hajj pilgrims in 2011. *Infectious Disorders - Drug Targets* 2014;14(2):110-16. doi: 10.2174/1871526514666141021112855
31. Choudhry A, Al Mudaimegh K, Turkistani A, et al. Hajj-associated acute respiratory infection among hajjis from Riyadh. *La Revue de Santé de la Méditerranée orientale* 2006;12(3/4):300-09.

32. Deris ZZ, Hasan H, Sulaiman SA, et al. The prevalence of acute respiratory symptoms and role of protective measures among Malaysian Hajj pilgrims. *Journal of Travel Medicine* 2010;17(2):82-88. doi: 10.1111/j.1708-8305.2009.00384.x
33. Emamian MH, Hassani AM, Fateh M. Respiratory tract infections and its preventive measures among Hajj pilgrims, 2010: A nested case control study. *International Journal of Preventive Medicine* 2013;4(9):1030-35.
34. Gautret P, Vu Hai V, Sani S, et al. Protective measures against acute respiratory symptoms in French pilgrims participating in the Hajj of 2009. *Journal of Travel Medicine* 2011;18(1):53-55. doi: 10.1111/j.1708-8305.2010.00480.x
35. Hashim S, Ayub ZN, Mohamed Z, et al. The prevalence and preventive measures of the respiratory illness among malaysian pilgrims in 2013 hajj season. *Journal of Travel Medicine* 2016;23(2) doi: 10.1093/jtm/tav019
36. Zein U. The role of using masks to reduce acute upper respiratory tract infections in pilgrims. 4th Asia Pacific travel health conference; 2002 Oct 20; Shanghai, PR China.
